# Planetary Health Diet Adherence in Korean Adults: Association with the Korean Healthy Eating Index

**DOI:** 10.3390/nu17193060

**Published:** 2025-09-25

**Authors:** Su-Jin Lee, Ji-Yun Hwang

**Affiliations:** 1 Department of Foodservice Management and Nutrition, Graduate School, Sangmyung University, Seoul 03016, Republic of Korea; juliasuj@naver.com; 2 Major of Foodservice Management and Nutrition, Sangmyung University, Seoul 03016, Republic of Korea

**Keywords:** sustainable diets, diet quality, healthy eating index

## Abstract

Background/Objectives: The Planetary Health Diet (PHD) was developed to address global health and environmental challenges by promoting sustainable and nutritionally adequate eating patterns. This study evaluated adherence to the PHD among Korean adults and examined its association with the Korean Healthy Eating Index (KHEI), with the aim of informing the development of a Korea-specific PHD adherence index. Methods: Using data from the 2013–2023 Korea National Health and Nutrition Examination Survey (KNHANES), dietary intake of adults aged ≥19 years was analyzed. Adherence was assessed by comparing food group consumption with PHD reference ranges, and KHEI scores were calculated to examine their association with PHD compliance and nutrient intake. Analyses were also stratified by sex to examine differences in intake patterns. Results: Men generally consumed larger quantities and had higher frequencies of intake across most food groups, whereas women consumed more fruits and dairy products. However, both sexes showed insufficient consumption of whole grains, legumes, and nuts, and red meat intake far exceeded the suggested limits. Participants with higher KHEI scores demonstrated greater intake of plant-based proteins and lower intake of red meat and saturated fats. Nutrient profiles also improved with higher KHEI scores. Conclusions: These findings suggest that better diet quality, as indicated by higher KHEI scores, is aligned with more sustainable eating behaviors and that that the KHEI may serve as a practical proxy for assessing adherence to the PHD. However, persistent gaps in whole grain, legume, and nut intake, together with excessive red meat consumption, highlight the need for culturally adapted guidelines and strategies to promote sustainable dietary shifts in Korea.

## 1. Introduction

As the need for dietary transformation to address climate change and global health crises is increasingly emphasized worldwide, the EAT-Lancet Commission proposed the Planetary Health Diet (PHD)—a dietary pattern designed to promote both human and planetary health [1]. The PHD recommends the consumption of vegetables, fruits, whole grains, and plant-based proteins, while limiting the intake of red and processed meats, refined grains, and foods high in added sugars, thereby aiming to achieve both health promotion and environmental sustainability [2]. However, the extent to which these recommendations align with the dietary patterns of the Korean population has yet to be clearly assessed.

Accordingly, various countries have developed and applied assessment tools to evaluate adherence to the PHD. For example, Brazil’s Planetary Health Diet Index (PHDI) consists of 16 components encompassing both recommended and limited food groups, quantifying dietary quality and sustainability [3]. The Planetary Health Diet Index for the United States (PHDI-US) incorporates features such as the proportion of ultra-processed foods and the balance between plant-based and animal-based food intake, reflecting national dietary characteristics [4]. The Planetary Health Diet Index for children and adolescents (PHDI-C), developed in Chile, was designed for use in younger populations and simplifies scoring based on the frequency of intake of major food groups [5]. Meanwhile, Italy’s Nutrient-Based EAT index (NB-EAT) was developed based on the PHD guidelines and evaluates whether individual food group intakes fall within the recommended ranges, using national dietary intake data to ensure practical applicability [6]. In addition to these, various countries are continuing to develop PHD-based indices tailored to their national contexts and dietary patterns, with a growing emphasis on integrating considerations for planetary sustainability [7,8,9,10,11,12].

Meanwhile, the Korean Healthy Eating Index (KHEI) is a tool based on data from the Korea National Health and Nutrition Examination Survey (KNHANES) to quantify the overall quality of dietary intake among Koreans [13]. It consists of 14 components that evaluate dietary behavior in terms of adequacy, moderation, and balance. The KHEI provides a comprehensive assessment of individual diet quality and allows for the quantification of health-related eating behaviors, making it a useful tool for examining the relationship between diet quality and adherence to the PHD among the Korean population.

Based on this background, the present study aims to evaluate the extent to which the Korean population adheres to the PHD and to provide foundational data for the development of a Korean-specific PHD assessment index. Using nationally representative KNHANES data from 2013–2023, this study assessed adherence to PHD food group recommendations based on intake levels and analyzed consumption frequency according to energy and protein criteria. In addition, food group and nutrient intakes were compared across KHEI levels to explore how overall diet quality influences adherence to the PHD and nutrient consumption. Importantly, analyses were stratified by sex to examine differences in dietary intake patterns between men and women.

This study is significant in that it provides empirical evidence on the degree to which Korean dietary patterns align with the PHD, thereby offering foundational data to inform dietary policies and educational strategies that promote both health and sustainability. Furthermore, the findings may serve as a valuable basis for the future development of a Korean-specific PHD adherence index and the establishment of a national health and nutrition monitoring system.

## 2. Materials and Methods

### 2.1. Design and Data Collection

This study utilized data from KNHANES, a nationwide survey designed to comprehensively assess the health behaviors, prevalence of chronic diseases, and dietary and nutritional status of the Korean population. First launched in 1998, the survey was conducted every three years until the 3rd period in 2005, after which it was transitioned to an annual rolling survey system starting from the 4th period (2007–2009). KNHANES consists of a health interview, a health examination, and a nutrition survey. The results are released annually in the form of press releases, statistical yearbooks, and publicly available raw data. Further information on the survey is available at the KNHANES website (https://knhanes.kdca.go.kr/, accessed on 20 September 2025).

This study analyzed data from KNHANES conducted between 2013 and 2023. The study population included adults aged 19 years and older who completed both the health examination and nutrition survey. Participants were excluded if they were pregnant or lactating, had been diagnosed with specific diseases by a physician, reported dietary intake that was not reflective of their usual consumption, or had extreme energy intake values (<500 kcal or >5000 kcal per day). Additionally, individuals with missing data on key variables such as smoking status, alcohol consumption, and household income were also excluded. The final number of participants included in the analysis is presented in Table 1.

All participants provided written informed consent, and the survey was conducted with approval from the Institutional Review Board (IRB) of the Korea Disease Control and Prevention Agency (KDCA) (2013-07CON-03-4C, 2013-12EXP-03-5C, 2018-01-03-P-A, 2018-01-03-C-A, 2018-01-03-2C-A, 2018-01-03-5C-A, 2018-01-03-4C-A, 2022-11-16-R-A). The 2015–2017 surveys were conducted without IRB approval, in accordance with the decision of the KDCA IRB that these years’ data collection activities did not fall under the scope requiring ethical review. The study was conducted according to the Declaration of Helsinki and approved by the Sangmyung University Institutional Review Board (IRB) (protocol code: IRB-SMU-S2024-1-005, approval date: 11 December 2024). The study was confirmed to be exempt from review in accordance with the Bioethics and Safety Act.

### 2.2. Methods and Variables

#### 2.2.1. Socio-Demographic Factors

In this study, the demographic and socioeconomic variables used in this study included sex, age, household income level, and education level. Sex was categorized as male or female, and age was grouped into 19–39, 40–64, and ≥65 years. Household income level was divided into four quartiles (low, low-middle, middle-high, and high), and education level was classified into elementary school or less, middle school, high school, and college or more.

#### 2.2.2. Assessment of Diet Intake

Dietary intake was assessed using 24-h recall data from the nutrition survey component of the KNHANES conducted between 2013 and 2023. Food group classification was based on the original survey variable (N_KINDG1), with further refinement using food code names when needed.

Whole grains and refined grains were classified based on product names: items explicitly labeled as whole grains (e.g., whole wheat, brown rice) were categorized as whole grains, while all others were classified as refined grains. For meat, beef, pork, lamb, and processed meats were grouped as red meat, while poultry such as chicken and duck were categorized as white meat. Seafood was classified as fish based on the Standard Tables of Fish and Shellfish Composition in Korea [14], published by the National Institute of Fisheries Science, using food code information.

Regarding fats, solid fats such as butter and margarine were classified as saturated fat, while liquid oils such as soybean oil and grape seed oil were categorized as unsaturated fat. For added sugars, processed foods were identified according to the *Manual for Determining Processed Foods* published by the Ministry of Food and Drug Safety (MFDS), and sugars derived from these processed foods were classified as added sugars [15]. Since data on sugar intake have been available only since 2016, analysis of total and added sugar intake was conducted using data from 2016 to 2023.

The number of servings for each food group was estimated by dividing the energy intake (kcal) of each group by the standard energy per serving (kcal) specified in the 2020 Dietary Reference Intakes for Koreans [16]. Specifically, servings were calculated using 300 kcal for grains, 100 kcal for meat, fish, eggs, and legumes, 15 kcal for vegetables, 50 kcal for fruits, 125 kcal for dairy products, and 45 kcal for fats and sugars. In addition, for protein-rich foods (e.g., legumes, nuts, meat, eggs, fish), protein-based servings were also calculated based on the amount of protein intake (g).

#### 2.2.3. Assessment of Diet Quality

In this study, diet quality was assessed using KHEI, a standardized evaluation tool developed for Korean adults [13]. The KHEI consists of 14 components that comprehensively reflect overall diet quality, grouped into three categories: adequacy, moderation, and balance.

The adequacy components assess whether individuals meet the recommended intake levels for key food groups based on national dietary guidelines. These include having breakfast regularly; consuming a variety of whole grains; ensuring sufficient intake of fruits (fresh, canned, or dried, excluding fruit juices); consuming fresh fruits separately; incorporating a wide range of vegetables, mushrooms, and seaweeds; eating non-salted vegetables distinct from kimchi or pickles, as per the guideline encouraging two to three types of non-kimchi vegetables per meal; and including meat, fish, eggs, legumes, as well as milk and dairy products in the daily diet. The moderation category includes the percentage of energy intake from saturated fatty acids, sodium intake, and the percentage of energy derived from beverages and added sugars. The balance category evaluates whether the energy contributions from carbohydrates and fats, as well as total energy intake, are within appropriate ranges.

Each component is scored based on predefined criteria, with the total score calculated out of 100 points. In the adequacy domain, frequency of breakfast intake and intake of meat, fish, eggs, and legumes are each scored from 0 to 10 points, while the remaining components are scored from 0 to 5 points, comprising a maximum of 55 points. The moderation domain includes three components—saturated fat intake ratio, sodium intake, and the energy contribution from beverages and added sugars—each scored from 0 to 10 points, for a total of 30 points. The balance domain includes energy intake, the percentage of energy from carbohydrates, and the percentage of energy from fat; each of these components is scored from 0 to 5 points, totaling 15 points.

The KHEI scoring criteria were developed in accordance with the 2015 Dietary Reference Intakes for Koreans (KDRI) [17]. As the index is calculated and provided only after the completion of raw data compilation for each KNHANES period, data from the 6th (2013–2015) to the 8th (2019–2021) periods have been made available. Therefore, this study utilized KHEI data from 2013 to 2021. Participants were categorized into quartiles (Q1–Q4) based on their total KHEI scores, with Q1 indicating the lowest diet quality and Q4 the highest. To enable comparison with the PHD recommendations, a mapping table linking PHD food groups and KHEI components was constructed and is provided as Supplementary Table S1.

### 2.3. Statistical Analysis

Data from the KNHANES conducted between 2013 and 2023 were combined and analyzed. Analyses were stratified by sex to examine differences in food group intake, consumption frequency, and nutrient intake between men and women. For continuous variables, descriptive statistics were calculated and presented as means and standard errors (SE), while categorical variables were summarized as frequencies and weighted percentages. Differences by sex were assessed using the Rao–Scott chi-square test for categorical variables and the PROC SURVEYREG procedure for continuous variables, with weights applied to account for the complex sampling design. For analyses across KHEI quartile groups, both food group and nutrient intakes were compared using PROC SURVEYREG, and linear trends were tested by treating the quartile categories as an ordinal variable (*p* for trend). All statistical analyses were performed using SAS version 9.4 (SAS Institute, Cary, NC, USA), and statistical significance was set at *p* < 0.05.

## 3. Results

### Socio-Demographic Factors According to Survey Period

Table 1 presents the socio-demographic characteristics of 12,129 participants by sex. Overall, 47.4% of participants were men and 52.6% were women. Age distribution differed significantly between men and women (*p* < 0.0001), with a higher proportion of men in the ≥65 years group compared with women (15.0% vs. 8.0%). Household income also showed significant sex differences (*p* = 0.0092), with women more likely to be in the high-income group compared with men (33.2% vs. 32.3%). In contrast, education level did not differ significantly by sex (*p* = 0.1854). These differences were assessed using the Rao–Scott chi-square test, accounting for the complex sampling design.

Table 2 presents the average daily intake of major food groups compared with the PHD-recommended ranges. Significant sex differences were observed in almost all food groups except for whole grains, nuts, and saturated fats, where intake levels were very low. For most food groups showing significant differences, men had higher intakes than women, including total grains, red meat, poultry, fish, legumes, and added sugars. In contrast, women had significantly higher intakes of fruits and milk/dairy products (*p* < 0.0001 for both).

When compared with the PHD recommendations, total grain intake tended to exceed the suggested range, whereas whole-grain intake remained far below the recommended levels in both sexes. Red meat consumption exceeded the PHD upper limit regardless of sex, whereas poultry intake was within the recommended range. Fish and seafood intake fell within the possible range for both men and women, although men consumed more than women. Egg intake exceeded the PHD recommended level in both sexes. Legume and nut intakes were substantially below the recommended levels and close to the lower bound of the possible range. Vegetable intake was significantly greater in men; men met the recommended level, whereas women’s intake fell short of the PHD target but remained within the possible range. Fruit intake was lower than the recommended level in both sexes, but also within the possible range. Milk and dairy products, as well as added fats and oils, were consistently below the recommended intake in both sexes. Added sugar intake slightly exceeded the PHD upper limit.

**Figure 1:**
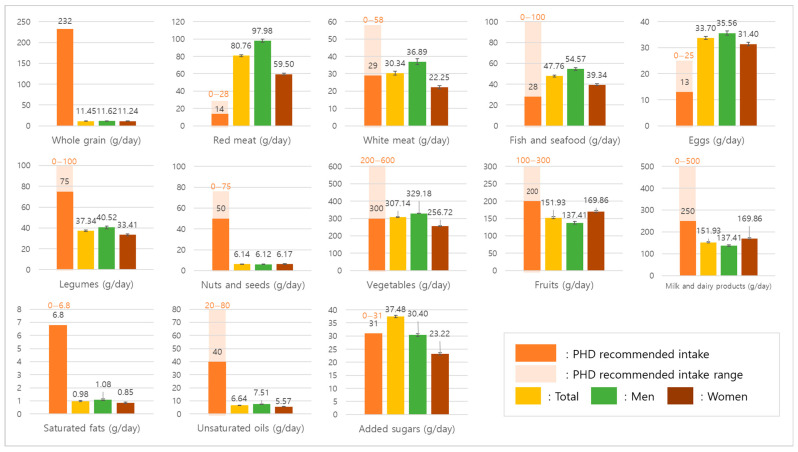
Average daily intake of major food groups compared with the PHD recommendations.

Figure 1 graphically illustrates the average daily intake of major food groups in comparison with the PHD reference intake and possible ranges, stratified by sex. The visualization highlights the marked discrepancies between actual consumption and the PHD targets as well as the sex-specific patterns observed in Table 2. In most food groups, men consumed larger amounts than women, including whole grains, red meat, white meat, fish, eggs, legumes, and added sugars. By contrast, women reported higher intakes of fruits and milk/dairy products. Whole grain and nut consumption remained very low in both sexes, and saturated fat intake showed no meaningful sex difference. When compared with the PHD recommendations, red meat intake exceeded the upper limit in both sexes, whereas vegetable intake in women and fruit intake in both sexes remained within the possible range despite falling short of the recommended levels. Milk and dairy products and unsaturated oils were consistently below the PHD reference across sexes, and egg intake exceeded the recommended level in both men and women.

Table 3 presents the energy-based frequency of food group consumption (servings/day) compared with the recommended intake levels from the KDRIs and the PHD. Significant sex differences were observed in all food groups except for nuts and seeds and saturated fats.

Whole-grain consumption frequency was extremely low in both sexes, far below both the KDRIs and PHD recommendations. Consequently, total grain consumption frequency was relatively high, indicating that refined grains accounted for most of the grain consumption, and men consumed more than women.

Red meat consumption frequency exceeded the PHD upper limit in both sexes, white meat frequency met the PHD recommendation in men but was below the target in women, while fish and seafood frequency exceeded the PHD recommendation in both sexes. Egg consumption frequency surpassed the PHD recommendation in both sexes, whereas legumes and nuts remained consistently below recommended levels, with no sex difference in nut consumption frequency.

Vegetable consumption frequency met the PHD recommendation in both sexes, but did not reach the KDRIs level. Fruit consumption frequency was below both the KDRIs and PHD recommendations in men and women alike.

Milk and dairy consumption frequency was substantially below both the KDRIs and PHD recommendations in both sexes, although women reported higher frequencies than men. Unsaturated oil consumption frequency remained below the PHD range, while saturated fat consumption frequency showed no meaningful sex difference. Added sugar consumption frequency was significantly higher in men than in women.

Table 4 presents the protein-based frequency of food group consumption (servings/day) compared with the recommended intake levels from the KDRIs and the PHD. Significant sex differences were found in all protein subgroups except for nuts and seeds.

Red meat consumption frequency was much higher than the PHD recommendation in both sexes, with men consuming more frequently than women. White meat frequency met the PHD target in both men and women. Fish and seafood were consumed at or above the PHD recommendation in both sexes, with higher frequencies in men. Egg frequency also exceeded the PHD guideline for both men and women, again with men consuming more frequently.

By contrast, legumes were consumed far less frequently than the levels recommended by either the KDRIs or PHD, and nut consumption was very low overall, showing no meaningful sex difference. When protein sources were considered as a whole, both men and women failed to meet the recommended levels of either the KDRIs or the PHD.

These findings suggest that while men consumed protein foods more frequently overall, both sexes showed substantial inadequacy in plant-based protein sources such as legumes and nuts, and total protein food frequency remained insufficient compared with dietary guidelines.

Table 5 presents food group intakes across KHEI quartiles. Participants in the highest quartile (Q4) showed significantly greater consumption of most PHD-recommended food groups, including whole grains, legumes, nuts and seeds, vegetables, and fruits (all *p* < 0.0001). In contrast, intake of red meat declined significantly as KHEI scores increased, while white meat also decreased but not significantly (*p* for trend = 0.4138 in men and 0.8582 in women). Fish and seafood, eggs, legumes, and nuts increased with higher KHEI levels.

Specifically, whole grain intake increased nearly five-fold from Q1 to Q4 but remained far below the PHD recommendation. Red meat intake decreased substantially across quartiles, though it still exceeded the PHD target. Conversely, fish and legume intake more than doubled from the lowest to the highest quartile. These findings indicate that individuals with higher KHEI scores tend to shift protein sources away from terrestrial animal meats toward plant-based and marine-based options, reflecting greater alignment with the core principles of the PHD.

Table 6 shows nutrient intake from foods according to KHEI quartiles. Total energy intake increased progressively from Q1 to Q4, accompanied by a rise in protein intake. Intakes of dietary fiber and key micronutrients, including vitamin A, vitamin C, calcium, potassium, and iron, also exhibited significant positive trends with higher KHEI scores (all; *p* < 0.0001). In contrast, total fat intake decreased across quartiles, with a marked reduction in saturated fatty acid intake as well. Monounsaturated fatty acid intake also showed downward trends in higher KHEI quartiles. These associations remained consistent when analyses were stratified by sex.

Table 7 shows the associations between healthy protein food intakes recommended in the PHD (fish, legumes, and nuts/seeds) and fatty acid consumption as well as KHEI scores.

For fish intake, total fat intake did not differ significantly across quartiles among men and women, but the composition of fatty acids showed clear trends. As fish intake increased, saturated fatty acid (SFA) and monounsaturated fatty acid (MUFA) intakes significantly decreased, whereas polyunsaturated fatty acid (PUFA) intake significantly increased in both sexes (all *p* for trend < 0.01). Higher fish intake was also positively associated with overall KHEI scores and the KHEI sub-score for the percentage of energy from SFA (all *p* for trend < 0.0001).

For legume intake, both men and women showed decreasing trends in saturated fatty acid (SFA) intake and increasing trends in polyunsaturated fatty acid (PUFA) intake with higher legume consumption. The trend for SFA was significant in men (*p* for trend = 0.0161) but not in women (*p* for trend = 0.1706), whereas PUFA intake increased significantly in both sexes (all *p* for trend < 0.0001). In addition, higher legume intake was associated with increasing overall KHEI scores in both men and women.

For nuts and seeds intake, participants with higher intakes (NQ4) showed significantly higher total fat, MUFA, and PUFA intakes compared to those with lower intakes (NQ1), in both men and women (all *p* for trend < 0.0001). KHEI scores and the sub-score for % energy from SFA also increased significantly with nut and seed consumption (all *p* for trend < 0.0001).

## 4. Discussion

This study assessed the extent to which Korean adults’ diets align with PHD recommendations using KNHANES data, aiming to inform the development of a Korean-specific PHD assessment index. Although notable gaps existed between current dietary patterns and PHD targets, individuals with higher KHEI scores tended to follow dietary patterns more consistent with PHD principles.

Overall, the results of this study demonstrate that the dietary patterns of Korean adults only partially align with the principles of the PHD. On the one hand, intakes of vegetables, fruits, and seafood were generally within or close to the recommended ranges, and individuals with higher KHEI scores showed greater adherence to these plant-forward components. On the other hand, the intake of whole grains, legumes, and nuts remained critically below target levels, while red meat and egg consumption exceeded the recommended upper limits. These findings suggest that although higher diet quality—as reflected by the KHEI—was associated with improved alignment with the PHD, substantial imbalances in specific food groups persist. Taken together, these patterns indicate that the Korean diet has not yet achieved a level consistent with the dual objectives of promoting human health and reducing environmental impact, highlighting the need for dietary modifications at both individual and policy levels.

However, despite this positive trend, imbalances in the consumption of specific food groups remain a significant barrier to achieving both health and environmental sustainability goals [18,19]. Whole grain intake was only a fraction of the PHD target, and legumes and nuts were consistently below recommended levels, reflecting the Korean diet’s continued reliance on refined grains and animal-based foods [20,21]. This rice-centered tradition limits whole grain consumption, making direct application of PHD-recommended targets challenging [22]. Nevertheless, a gradual shift from refined to whole grains, along with greater diversity and intake of plant-based foods, represents a culturally meaningful and necessary strategy to promote health and sustainability in Korea [20,23,24].

Culturally feasible strategies to achieve this include the gradual introduction of mixed grains (e.g., barley, millet, brown rice blends) into rice-based meals, particularly through public institutional cafeterias where incremental menu changes can normalize whole grain consumption. Evidence from a study of government worksite cafeteria users in Seoul indicates that affective attitudes and perceived behavioral control are key determinants of intention to adopt the PHD, and that acceptance depends not only on health benefits but also on taste, satiety, and menu appeal [25]. Therefore, providing diverse and palatable recipes, improving the workplace food environment, and strengthening organizational and social support may help overcome barriers and promote whole grain consumption in a culturally acceptable and sustainable manner.

While vegetable and fruit intake were largely within the PHD-recommended ranges, this trend cannot be viewed entirely positively. Although formal trend analyses were not conducted in this study, recent evidence indicates that fruit consumption in Korea has been steadily declining [26]. This downward shift is worrisome, as substantial evidence has shown that sufficient intake of vegetables and fruits plays a critical role in reducing the risk of chronic diseases such as metabolic syndrome and cardiovascular disease [27,28,29].

The decline in fruit consumption observed in this study may not be solely attributable to individual dietary behaviors but is likely influenced by broader economic factors, including rising fruit prices driven by climate change, distribution costs, and reduced domestic yields [30]. The increasing reliance on inexpensive imported fruits, such as bananas, further reflects these economic pressures, yet their long-distance transportation contributes to higher greenhouse gas emissions, raising environmental concerns [31]. Thus, strategies to promote fruit intake should emphasize not only greater consumption overall but also the selection of seasonal and locally produced fruits to reduce the dietary carbon footprint [32], consistent with the recommendations of the PHD framework.

Moreover, beyond vegetables and fruits, the results also indicate that dairy and oils remained within or below the ranges proposed by the PHD. Specifically, dairy consumption in Korea was consistently lower than both the PHD reference intake and the KDRIs, reflecting a long-standing pattern of limited dairy intake in the Korean diet. Likewise, oils and fats were maintained within the suggested range. Added sugar intake slightly exceeded the recommended threshold; however, in this study, the amount of sugar was estimated based on sucrose contained in processed foods rather than true added sugars. As such, the current approach may have led to an overestimation, highlighting the need for a more refined added sugar database in future research.

Additionally, our findings indicate that improvements in the consumption patterns of animal-based foods are warranted. In this study, red meat intake remained well above the PHD upper limit, and although individuals with higher KHEI scores tended to consume slightly less, overall intake levels were still excessive. Egg consumption also showed a steady upward trend across survey cycles, surpassing the recommended limits of both the PHD and the KDRIs. While total seafood intake was generally aligned with PHD targets, fish consumption specifically exhibited a declining pattern and fell short of the minimum recommended threshold, underscoring the need for strategies to encourage greater fish intake and improve access to a diverse range of healthy protein sources.

Nevertheless, when dietary patterns were examined by KHEI score groups, a more favorable shift became evident. The results from Table 5 further support the interpretive value of KHEI in assessing alignment with the PHD. Specifically, the intake of fish, legumes, and nuts—key food groups emphasized in the PHD for their health and sustainability benefits—increased consistently from the lowest KHEI score group (Q1) to the highest (Q4). Participants with higher KHEI scores (Q4) consumed significantly greater amounts of these plant- and seafood-based protein sources compared with those with lower scores (Q1). This trend underscores that higher diet quality, as captured by the KHEI, is associated not only with reduced red meat intake but also with a gradual shift toward more sustainable protein choices. Such findings reinforce the dual role of KHEI in reflecting both nutritional adequacy and environmental sustainability, and they highlight its potential as a basis for developing a Korean-specific PHD adherence index.

As emphasized in previous research [33,34,35,36], a gradual reduction in red meat consumption is essential for promoting both human and planetary health. Simultaneously, shifting toward white meat, such as poultry, which imposes a comparatively lower environmental burden, should be encouraged. To address the protein gap in a sustainable manner, increasing the intake of plant-based protein sources—including legumes and nuts—is critical. Supporting this dietary transition requires continuous public education and awareness-raising efforts that help individuals understand the environmental and nutritional differences among various protein sources [37,38,39]. Such initiatives are key to fostering dietary patterns aligned with the principles of the PHD.

Participants with higher KHEI scores exhibited distinct nutrient intake patterns. As shown in Table 6, individuals in the highest quartile of KHEI had greater total energy and protein intakes, alongside higher consumption of essential micronutrients such as vitamin C, calcium, and potassium. Conversely, their total fat and saturated fatty acid intakes were significantly lower than those of individuals in the lowest quartile. These findings suggest that higher diet quality, as captured by the KHEI, is not only associated with improved nutrient adequacy but also with a favorable macronutrient distribution profile that may contribute to reduced chronic disease risk.

In addition, the analysis of food group intakes presented in Table 7 provides further insights into the relationship between healthy protein sources, dietary fat quality, and overall diet quality. Higher quartiles of fish intake were significantly associated with lower saturated fatty acid intake and higher KHEI scores in both men and women, despite total fat intake remaining relatively stable across quartiles. This pattern suggests that greater fish consumption may contribute to improved fat quality rather than increasing overall fat intake. Similarly, higher intakes of legumes were positively associated with higher KHEI scores and reduced saturated fat consumption, underscoring their role as a plant-based protein source that supports both health and sustainability. Nuts and seeds intake also showed a consistent positive association with KHEI scores, accompanied by favorable shifts in fatty acid profiles, including increased polyunsaturated fatty acids. Collectively, these findings indicate that individuals adhering to higher-quality diets are more likely to incorporate a variety of protein sources emphasized in the PHD, which not only enhances nutrient adequacy but also promotes a healthier lipid profile.

This study suggests that the KHEI is not only an index for assessing dietary quality but also holds interpretive value in evaluating alignment with the global standard for sustainable diets, as represented by the PHD. The KHEI encompasses key elements consistent with the PHD—such as balanced energy intake, higher consumption of vegetables, fruits, and whole grains, and reduced intake of sodium and saturated fats—while also including unique components like breakfast consumption and sodium restriction. As shown in Supplementary Table S1, many PHD food groups directly or partially match KHEI components, suggesting that the KHEI provides a solid foundation for developing a Korean-specific PHD index. By incorporating additional PHD-specific indicators, such as plant-based protein intake and environmental sustainability, the KHEI framework could evolve into a comprehensive national monitoring tool for sustainable dietary practices.

Cross-national studies reveal that low adherence to the PHD and imbalances in key food groups are common across diverse contexts. In Japan, excessive red meat intake relative to the PHD was observed, particularly in younger and middle-aged adults, though overall protein intake remained within national recommendations [40]. In the Netherlands, PHD-modeled diets emphasized vegetables, legumes, and nuts but showed insufficient calcium and several B vitamins, echoing concerns in Korea, where dairy consumption is relatively low [41]. In Portugal, adherence to the PHD was generally poor, with excessive meat and added sugar and inadequate intake of pulses, nuts, and whole grains; socioeconomic inequalities played an important role in shaping these patterns [42]. In Australia, average diets were characterized by very high consumption of discretionary foods and red meat, and inadequate intake of vegetables, cereals, and plant-based alternatives [43]. When compared with our findings in Korea, these results underscore that while the PHD provides a valuable global benchmark, its adoption requires culturally sensitive adaptations and targeted strategies tailored to national dietary traditions and nutritional vulnerabilities.

Moreover, international studies emphasize the importance of considering factors not fully addressed in the original PHD framework—such as age-specific nutrient requirements, socioeconomic disparities, and fluid intake guidelines—while advocating for multidimensional and country-specific approaches [44]. In this context, a Korean-adapted PHD should reflect national food culture, nutrient profiles, environmental goals, and social determinants of health. The successful development and implementation of such a model would require the support of integrated strategies involving nutrition policy reform, education campaigns, and systemic improvements in the food environment.

Nevertheless, this study has several limitations. While the link between diet quality and sustainability is important, we did not directly assess associations between PHD/KHEI adherence and health outcomes such as BMI, blood lipids, metabolic syndrome, or cardiovascular risk markers. In addition, due to the structure of the KNHANES food grouping system, some food items could not be perfectly aligned with the PHD reference categories, such as distinguishing refined from whole grains or processed from unprocessed meats. Although data from multiple survey cycles were analyzed, we did not conduct a formal trend analysis across years, which may have provided additional insights into temporal changes in dietary patterns. Finally, while the PHD emphasizes both human and planetary health, our analysis was restricted to dietary quality indicators and did not directly incorporate environmental measures such as greenhouse gas emissions or land use. Future research should address these limitations to provide a more comprehensive evaluation of sustainable diets in the Korean context.

## 5. Conclusions

This study evaluated the alignment between Korean adults’ dietary patterns and the PHD framework using KNHANES data and found that higher KHEI scores were positively associated with greater adherence to PHD principles. These findings suggest that KHEI, as an established tool for assessing diet quality, could serve as a useful foundation for evaluating sustainable diets in Korea. Nonetheless, substantial gaps remain in the intake of whole grains, legumes, and nuts, while red meat consumption continues to exceed recommended levels. These imbalances underline the need to develop a Korean-specific PHD index that reflects cultural dietary practices while promoting health and environmental sustainability.

## Figures and Tables

**Table 1:** Socio-Demographic Characteristics by KNHANES Survey Period (2013–2023).

Characteristics	Total	Men	Women	*p*-Value
Total	12,129 (100.0)	5744 (47.4)	6385 (52.6)	-
Age (yr.)				<0.0001
19~39 y	4350 (35.9)	2040 (35.5)	2310 (36.2)	
40~64 y	6406 (52.8)	2843 (49.5)	3563 (55.8)	
≥65 y	1373 (11.3)	861 (15.0)	512 (8.0)	
Household income				0.0092
Low	1371 (11.3)	708 (12.3)	663 (10.4)	
Low middle	2942 (24.3)	1386 (24.1)	1556 (24.4)	
Middle-high	3841 (31.7)	1794 (31.2)	2047 (32.1)	
High	3975 (32.8)	1856 (32.3)	2119 (33.2)	
Education level				0.1854
Elementary school or less	1115 (9.2)	544 (9.5)	571 (8.9)	
Middle school	908 (7.5)	453 (7.9)	455 (7.1)	
High school	4576 (37.7)	2174 (37.9)	2402 (37.6)	
College or more	5530 (45.6)	2573 (44.8)	2957 (46.3)	

KNHANES, Korea National Health and Nutrition Examination Survey. Values are presented as an unweighted number (n) and a weighted percentage (%). *p*-values were obtained using the Rao–Scott chi-square test.

**Table 2:** Average Food Group Intake by KNHANES Survey Period (2013–2023).

Components		PHD (Possible Range)	Total	Men (n = 5744)	Women (n = 6385)	*p*-Value
Whole grains		232	11.45 ± 0.31	11.62 ± 0.44	11.24 ± 0.37	0.5046
Total grains		-	320.12 ± 1.95	322.41 ± 2.41	239.29 ± 1.92	<0.0001
Red meat (beef, pork, etc.)		14 (0–28)	80.76 ± 1.24	97.98 ± 1.91	59.50 ± 1.19	<0.0001
White meat (poultry)		29 (0–58)	30.34 ± 1.03	36.89 ± 1.67	22.25 ± 0.97	<0.0001
Fish and seafood		28 (0–100)	47.76 ± 0.85	54.57 ± 1.30	39.34 ± 0.92	<0.0001
Eggs		13 (0–25)	33.70 ± 0.57	35.56 ± 0.81	31.40 ± 0.68	<0.0001
Legumes (beans, soy)		75 (0–100)	37.34 ± 0.88	40.52 ± 1.35	33.41 ± 1.04	<0.0001
Nuts and seeds		50 (0–75)	6.14 ± 0.27	6.12 ± 0.35	6.17 ± 0.36	0.9340
Protein sources		-	236.03 ± 1.98	271.64 ± 2.93	192.07 ± 2.15	<0.0001
Vegetables		300 (200–600)	307.14 ± 2.41	329.18 ± 3.35	256.72 ± 2.81	<0.0001
Fruits		200 (100–300)	151.93 ± 2.80	137.41 ± 3.68	169.86 ± 3.70	<0.0001
Milk and dairy products		250 (0–500)	87.58 ± 1.69	81.63 ± 2.39	94.92 ± 2.27	<0.0001
Added fats/oils	Saturated fats	6.8 (0–6.8)	0.98 ± 0.04	1.08 ± 0.06	0.85 ± 0.05	0.0029
	Unsaturated oils	40 (20–80)	6.64 ± 0.09	7.51 ± 0.14	5.57 ± 0.10	<0.0001
Added sugars (n = 8725)		31 (0–31)	37.48 ± 0.41	30.40 ± 0.54	23.22 ± 0.39	<0.0001

KNHANES: Korea National Health and Nutrition Examination Survey. PHD: Planetary Health Diet. Values are presented as mean ± standard error (SE). *p*-values were obtained using complex sample analysis methods with sampling weights applied. *p*-values were adjusted for age, household income, and education level. Added sugar intake was analyzed only from KNHANES VII–IX (2016–2023), as data were not disclosed in KNHANES VI (2013–2015); thus, the total sample size was 8725 participants (4137 men and 4588 women).

**Table 3:** Energy-Based Frequency of Food Group Intake by KNHANES Survey Period (2013–2023).

Components		KDRIs		PHD		Total	Men (n = 5744)	Women (n = 6385)	*p*-Value
		2000	2500	2000	2500				
Whole grains		3.5	4	2.5	3	0.13 ± 0.004	0.14 ± 0.005	0.13 ± 0.004	<0.0001
Total grains						3.27 ± 0.018	3.55 ± 0.025	2.56 ± 0.019	<0.0001
Red meat (beef, pork, etc.)				0.2	0.3	1.86 ± 0.032	2.25 ± 0.050	1.39 ± 0.030	<0.0001
White meat (poultry)				0.5	0.6	0.50 ± 0.018	0.60 ± 0.030	0.36 ± 0.017	<0.0001
Fish and seafood				0.3	0.4	0.63 ± 0.011	0.72 ± 0.017	0.52 ± 0.011	<0.0001
Eggs				0.2	0.2	0.52 ± 0.009	0.55 ± 0.013	0.49 ± 0.011	<0.0001
Legumes (beans, soy)				2.3	2.8	0.43 ± 0.008	0.46 ± 0.012	0.38 ± 0.010	<0.0001
Nuts and seeds				2.3	2.9	0.21 ± 0.006	0.21 ± 0.009	0.21 ± 0.008	0.7418
Protein sources		4	5	6	7.5	4.15 ± 0.039	4.80 ± 0.059	3.33 ± 0.038	<0.0001
Vegetables		8	8	4	5	6.41 ± 0.047	7.15 ± 0.070	5.50 ± 0.055	<0.0001
Fruits		2	4	2	2.5	1.54 ± 0.028	1.38 ± 0.035	1.74 ± 0.038	<0.0001
Milk and dairy products		1	1	1	1	0.58 ± 0.011	0.54 ± 0.016	0.63 ± 0.015	<0.0001
Added fats/oils	Unsaturated oils	4	7	10	13	1.35 ± 0.019	1.53 ± 0.028	1.13 ± 0.019	<0.0001
	Saturated fats					0.12 ± 0.005	0.13 ± 0.007	0.10 ± 0.006	0.0426
Added sugars (n = 8725)						0.83 ± 0.009	0.94 ± 0.014	0.70 ± 0.010	<0.0001

KDRIs: Korean Dietary Reference Intakes. KNHANES: Korea National Health and Nutrition Examination Survey. PHD: Planetary Health Diet. Values are presented as mean ± standard error (SE). Values represent the energy-based frequency of food group intake (servings/day). *p*-values were obtained using complex sample analysis methods with sampling weights applied and were adjusted for age, household income, and education level. Added sugar intake was analyzed only from KNHANES VII–IX (2016–2023), as data were not disclosed in KNHANES VI (2013–2015); thus, the total sample size was 8725 participants (4137 men and 4588 women).

**Table 4:** Protein-Based Frequency of Food Group Intake by KNHANES Survey Period (2013–2023).

Components	KDRIs		PHD		Total	Men (n = 5744)	Women (n = 6385)	*p*-Value
	2000	2500	2000	2500				
Red meat (beef, pork, etc.)			0.2	0.3	1.48 ± 0.022	1.82 ± 0.035	1.07 ± 0.020	<0.0001
White meat (poultry)			0.5	0.6	0.69 ± 0.024	0.85 ± 0.039	0.51 ± 0.022	<0.0001
Fish and seafood			0.3	0.4	0.92 ± 0.016	1.05 ± 0.024	0.75 ± 0.016	<0.0001
Eggs			0.2	0.2	0.45 ± 0.008	0.47 ± 0.011	0.42 ± 0.009	<0.0001
Legumes (beans, soy)			2.3	2.8	0.35 ± 0.007	0.39 ± 0.010	0.32 ± 0.008	<0.0001
Nuts and seeds			2.3	2.9	0.07 ± 0.002	0.08 ± 0.004	0.07 ± 0.003	0.2751
Protein sources	4	5	6	7.5	3.97 ± 0.035	4.65 ± 0.053	3.13 ± 0.034	<0.0001

KDRIs: Korean Dietary Reference Intakes. KNHANES: Korea National Health and Nutrition Examination Survey. PHD: Planetary Health Diet. Values are presented as mean ± standard error (SE). *p*-values were obtained using complex sample analysis methods with sampling weights applied. *p*-values were adjusted for age, household income, and education level.

**Table 5:** Food Group Intake by KHEI Quartiles in Comparison with PHD.

Components		PHD (Possible Range)	Men (n = 4500)					Women (n = 4924)				
			Q1 (n = 1252)	Q2 (n = 1168)	Q3 (n = 1136)	Q4 (n = 944)	*p* for Trend	Q1 (n = 1104)	Q2 (n = 1188)	Q3 (n = 1220)	Q4 (n = 1412)	*p* for Trend
Whole grains		232	4.82 ±0.60	8.97 ±0.76	16.04 ±1.11	24.62 ±1.57	<0.0001	4.22 ±0.49	9.02 ±0.79	13.29 ±0.93	20.75 ±0.99	<0.0001
Total grains		-	304.38 ±5.47	329.32 ±5.44	360.77 ±5.24	338.34 ±4.76	<0.0001	221.17 ±4.68	244.85 ±4.57	260.91 ±4.10	264.00 ±3.62	<0.0001
Red meat (beef, pork, etc.)		14 (0–28)	115.24 ±4.60	92.56 ±3.71	87.35 ±3.58	78.76 ±2.88	<0.0001	65.49 ±3.18	61.77 ±2.76	56.00 ±2.48	50.68 ±1.81	0.0140
White meat (poultry)		29 (0–58)	40.02 ±3.90	33.63 ±3.74	34.86 ±3.33	38.26 ±3.84	0.4138	23.27 ±2.76	24.41 ±2.36	21.43 ±1.89	20.42 ±1.79	0.8582
Fish and seafood		28 (0–100)	40.06 ±2.45	56.59 ±3.30	66.19 ±3.02	69.83 ±2.96	<0.0001	27.82 ±2.58	39.13 ±2.14	43.59 ±1.86	49.19 ±2.05	<0.0001
Eggs		13 (0–25)	29.17 ±1.73	31.34 ±1.64	37.04 ±1.71	43.64 ±2.07	<0.0001	23.13 ±1.42	27.35 ±1.36	32.74 ±1.66	34.92 ±1.36	<0.0001
Legumes (beans, soy)		75 (0–100)	23.47 ±3.46	33.27 ±2.14	48.19 ±2.78	63.87 ±3.75	<0.0001	18.28 ±1.51	28.07 ±2.51	32.47 ±2.72	46.68 ±2.38	<0.0001
Nuts and seeds		50 (0–75)	2.83 ±0.39	3.83 ±0.41	8.93 ±1.14	10.17 ±1.18	<0.0001	2.91 ±0.44	4.23 ±0.54	7.72 ±0.90	10.40 ±1.24	<0.0001
Protein sources		-	250.79 ±7.43	251.24 ±5.89	282.55 ±5.52	304.54 ±5.89	<0.0001	160.89 ±5.34	184.97 ±4.85	193.95 ±4.67	212.28 ±3.71	<0.0001
Vegetables		300 (200–600)	255.06 ±5.87	336.39 ±7.06	382.07 ±7.57	401.76 ±7.87	<0.0001	176.67 ±4.77	239.90 ±5.79	287.30 ±6.41	331.05 ±6.30	<0.0001
Fruits		200 (100–300)	53.31 ±4.46	109.53 ±8.74	176.20 ±8.68	289.67 ±10.92	<0.0001	67.17 ±5.63	138.38 ±9.35	193.98 ±8.71	290.46 ±8.08	<0.0001
Milk and dairy products		250 (0–500)	49.24 ±4.38	74.15 ±5.13	79.16 ±5.26	137.51 ±6.05	<0.0001	66.56 ±5.13	74.92 ±4.74	86.44 ±4.80	142.71 ±4.53	<0.0001
Added fats/oils	Saturated fats	6.8 (0–6.8)	1.52 ±0.18	1.00 ±0.13	1.29 ±0.14	1.06 ±0.12	0.0583	1.44 ±0.17	0.83 ±0.11	0.91 ±0.11	0.83 ±0.12	0.0694
	Unsaturated oils	40 (20–80)	6.70 ±0.31	7.82 ±0.37	7.75 ±0.27	8.15 ±0.29	<0.0001	4.63 ±0.24	5.27 ±0.20	5.93 ±0.22	6.63 ±0.23	<0.0001
Added sugars		31 (0–31)	36.19 ±1.33	28.37 ±1.10	23.86 ±0.97	20.69 ±1.01	<0.0001	27.33 ±1.07	22.15 ±0.90	19.47 ±0.77	16.76 ±0.67	<0.0001

KHEI, Korean Healthy Eating Index; PHD, Planetary Health Diet. Values are presented as mean ± standard error (SE). *p*-values were obtained from survey-weighted regression models adjusted for age, household income, and education, with sampling weights applied. Quartiles of KHEI were defined from the overall distribution and applied separately for men and women.

**Table 6:** Nutrient intakes across quartiles of KHEI scores, by sex.

Components	Men (n = 4500)					Women (n = 4924)				
	Q1 (n = 1252)	Q2 (n = 1168)	Q3 (n = 1136)	Q4 (n = 944)	*p* for Trend	Q1 (n = 1104)	Q2 (n = 1188)	Q3 (n = 1220)	Q4 (n = 1412)	*p* for Trend
Total energy (kcal)	2222.86 ±30.95	2254.78 ±27.23	2402.47 ±25.81	2404.15 ±22.84	<0.0001	1519.83 ±23.87	1607.52 ±20.31	1735.23 ±18.74	1853.23 ±14.47	<0.0001
Total carbohydrate (g)	291.72 ±3.89	328.23 ±4.00	362.56 ±3.96	370.38 ±3.64	<0.0001	211.60 ±3.30	244.02 ±3.31	273.75 ±3.13	296.58 ±2.66	<0.0001
Dietary fiber (g)	20.83 ±0.40	25.07 ±0.41	29.66 ±0.50	33.18 ±0.47	<0.0001	15.45 ±0.29	19.69 ±0.35	23.70 ±0.38	28.22 ±0.35	<0.0001
Total protein (g)	77.15 ±1.38	81.51 ±1.30	88.75 ±1.23	93.18 ±1.28	<0.0001	51.66 ±1.05	58.11 ±0.91	62.29 ±0.85	69.14 ±0.66	<0.0001
Total fat (g)	62.55 ±1.52	50.66 ±1.04	50.69 ±0.90	51.92 ±0.74	<0.0001	43.99 ±1.18	39.53 ±0.84	39.79 ±0.76	41.43 ±0.52	0.0014
Saturated fatty acid (g)	20.77 ±0.51	15.78 ±0.35	14.88 ±0.29	15.03 ±0.25	<0.0001	15.11 ±0.45	12.53 ±0.31	11.98 ±0.26	12.21 ±0.17	<0.0001
Monounsaturated fatty acid (g)	21.44 ±0.61	16.25 ±0.39	15.85 ±0.32	16.18 ±0.27	<0.0001	14.36 ±0.44	12.67 ±0.29	12.53 ±0.27	12.88 ±0.18	0.0230
Polyunsaturated fatty acid (g)	13.48 ±0.36	12.83 ±0.30	13.74 ±0.28	14.48 ±0.26	<0.0001	9.56 ±0.27	9.65 ±0.23	10.63 ±0.24	11.35 ±0.20	<0.0001
Cholesterol (g)	281.01 ±8.10	280.12 ±7.70	306.56 ±7.96	337.02 ±8.93	<0.0001	202.10 ±6.67	219.20 ±6.21	230.56 ±6.45	252.92 ±5.25	<0.0001
Vitamin A (ug RAE)	350.95 ±15.54	411.97 ±15.70	494.14 ±32.20	496.36 ±11.71	<0.0001	282.78 ±10.65	331.05 ±10.36	386.07 ±14.20	448.89 ±10.82	<0.0001
Vitamin C (mg)	57.15 ±4.50	66.51 ±2.84	82.55 ±3.35	108.34 ±3.71	<0.0001	44.78 ±2.07	59.87 ±2.51	82.60 ±3.58	110.20 ±3.91	<0.0001
Vitamin B1 (mg)	1.59 ±0.04	1.70 ±0.03	1.92 ±0.03	2.06 ±0.03	<0.0001	1.04 ±0.03	1.22 ±0.02	1.38 ±0.02	1.61 ±0.03	<0.0001
Vitamin B2 (mg)	1.69 ±0.04	1.65 ±0.03	1.75 ±0.03	1.90 ±0.03	<0.0001	1.17 ±0.02	1.28 ±0.02	1.37 ±0.02	1.55 ±0.02	<0.0001
Niacin (mg)	15.31 ±0.31	15.83 ±0.30	17.88 ±0.34	18.97 ±0.32	<0.0001	10.27 ±0.24	11.89 ±0.21	12.79 ±0.19	14.42 ±0.18	<0.0001
Calcium (mg)	447.34 ±8.52	523.04 ±9.77	581.28 ±9.74	671.34 ±11.45	<0.0001	355.35 ±7.79	414.91 ±9.01	462.39 ±7.09	568.19 ±8.32	<0.0001
Sodium (mg)	4064.28 ±73.27	4253.08 ±73.82	4429.20 ±77.39	4139.53 ±72.82	0.0024	2768.30 ±75.25	2959.15 ±55.22	3112.07 ±53.56	3099.68 ±46.96	0.0002
Potassium (mg)	1055.69 ±16.33	1168.03 ±15.68	1315.46 ±15.51	1449.37 ±16.77	<0.0001	749.50 ±12.55	870.29 ±12.52	967.01 ±11.02	1139.54 ±10.10	<0.0001
Iron (mg)	11.99 ±0.28	14.56 ±0.35	16.33 ±0.33	17.68 ±0.34	<0.0001	8.24 ±0.19	10.66 ±0.23	12.12 ±0.21	14.31 ±0.22	<0.0001

KHEI, Korean Healthy Eating Index; RAE, retinol activity equivalents. Values are presented as mean ± SE. Nutrient intakes were adjusted for age, household income, and education. *p* for trend was calculated using linear regression across quartiles of KHEI scores within each sex group.

**Table 7:** Healthy Protein Food Intakes Recommended in the PHD and Their Associations with Fatty Acid Consumption and KHEI Scores.

Components (Quartiles)		Total Fat (g)	SFA (g)	MUFA (g)	PUFA (g)	KHEI Score	KHEI Sub-Score: % Energy from SFA
Fish Intakes							
Men	FQ1 (n = 1398)	56.85 ± 1.23	18.52 ± 0.43	19.24 ± 0.49	12.95 ± 0.31	54.51 ± 0.49	6.15 ± 0.16
	FQ2 (n = 1312)	53.04 ± 1.05	16.90 ± 0.37	17.54 ± 0.42	12.45 ± 0.24	58.62 ± 0.46	7.05 ± 0.15
	FQ3 (n = 1370)	53.48 ± 1.05	16.60 ± 0.36	17.39 ± 0.41	13.28 ± 0.27	61.80 ± 0.43	7.57 ± 0.13
	FQ4 (n = 1664)	55.63 ± 0.88	16.27 ± 0.28	17.31 ± 0.32	15.74 ± 0.28	63.26 ± 0.38	8.16 ± 0.10
*p* for trend		0.6479	<0.0001	0.0021	<0.0001	<0.0001	<0.0001
Women	FQ1 (n = 1634)	43.16 ± 0.82	14.37 ± 0.31	14.13 ± 0.30	9.91 ± 0.21	57.96 ± 0.48	5.93 ± 0.15
	FQ2 (n = 1720)	40.04 ± 0.71	12.74 ± 0.27	12.96 ± 0.26	9.66 ± 0.17	61.89 ± 0.41	7.01 ± 0.14
	FQ3 (n = 1663)	40.02 ± 0.68	12.37 ± 0.24	12.68 ± 0.24	10.38 ± 0.20	63.63 ± 0.42	7.37 ± 0.13
	FQ4 (n = 1368)	43.98 ± 0.83	13.03 ± 0.29	13.47 ± 0.28	12.41 ± 0.26	65.73 ± 0.45	7.85 ± 0.13
*p* for trend		0.9989	0.0001	0.0183	<0.0001	<0.0001	<0.0001
Legumes Intakes							
Men	LQ1 (n = 1841)	55.09 ± 0.97	18.12 ± 0.34	18.43 ± 0.37	12.32 ± 0.26	55.16 ± 0.39	6.45 ± 0.13
	LQ2 (n = 816)	55.05 ± 1.44	17.17 ± 0.50	17.98 ± 0.56	13.73 ± 0.36	58.30 ± 0.55	7.08 ± 0.18
	LQ3 (n = 1519)	53.46 ± 1.04	16.51 ± 0.35	17.45 ± 0.39	13.25 ± 0.27	61.39 ± 0.44	7.55 ± 0.13
	LQ4 (n = 1568)	55.75 ± 0.93	16.11 ± 0.31	17.48 ± 0.36	16.00 ± 0.27	65.07 ± 0.39	8.24 ± 0.11
*p* for trend		0.0112	0.0161	0.8524	<0.0001	<0.0001	<0.0001
Women	LQ1 (n = 2331)	41.55 ± 0.64	13.74 ± 0.25	13.50 ± 0.23	9.50 ± 0.16	58.49 ± 0.36	6.40 ± 0.13
	LQ2 (n = 1077)	40.37 ± 0.88	12.99 ± 0.32	12.85 ± 0.30	9.90 ± 0.25	61.77 ± 0.50	7.01 ± 0.17
	LQ3 (n = 1513)	40.71 ± 0.77	12.49 ± 0.27	13.13 ± 0.29	10.38 ± 0.21	64.34 ± 0.44	7.58 ± 0.13
	LQ4 (n = 1464)	43.97 ± 0.84	12.85 ± 0.29	13.48 ± 0.29	12.84 ± 0.25	66.87 ± 0.46	7.51 ± 0.14
*p* for trend		0.0007	0.1706	0.1836	<0.0001	<0.0001	<0.0001
Nuts and Seed Intakes							
Men	NQ1 (n = 1417)	51.76 ± 1.15	17.36 ± 0.43	17.06 ± 0.44	11.60 ± 0.27	54.81 ± 0.49	6.52 ± 0.15
	NQ2 (n = 1386)	49.85 ± 0.96	16.04 ± 0.33	16.19 ± 0.37	11.86 ± 0.24	59.37 ± 0.44	7.33 ± 0.13
	NQ3 (n = 1508)	56.22 ± 0.97	17.24 ± 0.34	18.22 ± 0.38	14.28 ± 0.25	61.10 ± 0.41	7.63 ± 0.13
	NQ4 (n = 1433)	61.63 ± 1.10	17.56 ± 0.35	20.04 ± 0.43	17.18 ± 0.35	63.97 ± 0.45	7.65 ± 0.13
*p* for trend		<0.0001	0.0322	<0.0001	<0.0001	<0.0001	<0.0001
Women	NQ1 (n = 1615)	39.39 ± 0.81	13.42 ± 0.31	12.71 ± 0.30	8.79 ± 0.20	57.16 ± 0.43	6.21 ± 0.15
	NQ2 (n = 1646)	37.48 ± 0.64	12.14 ± 0.24	11.85 ± 0.22	9.12 ± 0.18	61.30 ± 0.44	7.17 ± 0.13
	NQ3 (n = 1525)	43.28 ± 0.79	13.55 ± 0.30	13.67 ± 0.28	11.00 ± 0.21	63.45 ± 0.43	7.27 ± 0.13
	NQ4 (n = 1599)	47.29 ± 0.77	13.49 ± 0.25	15.17 ± 0.27	13.42 ± 0.25	67.46 ± 0.42	7.48 ± 0.13
*p* for trend		<0.0001	0.0137	<0.0001	<0.0001	<0.0001	<0.0001

SFA, saturated fatty acids; MUFA, monounsaturated fatty acids; PUFA, polyunsaturated fatty acids; KHEI, Korean Healthy Eating Index. Intakes of fish, legumes, and nuts and seeds were divided into sex-specific quartiles (Q1–Q4). Values are presented as mean ± SE and adjusted for age, household income, and education. *p* for trend was calculated by modeling the median value of each quartile as a continuous variable.

## Data Availability

The data are available from the Korean National Health and Nutrition Examination Survey (KNHANES) website at https://knhanes.kdca.go.kr/knhanes/main.do (accessed on 25 July 2025).

## Supplementary Materials

The following supporting information can be downloaded at: https://www.mdpi.com/article/10.3390/nu17193060/s1, Supplement Table S1: Matching table between PHD food groups and KHEI components.
