# Supplementary material for: Planetary Health Diet Adherence in Korean Adults: Association with the Korean Healthy Eating Index
Source: Nutrients. 2025 Sep 25;17(19):3060. doi: 10.3390/nu17193060 (PMC12525963; doi:10.3390/nu17193060)
Supplement: Supplementary file 1 [file nutrients-17-03060-s001.zip › nutrients-3863828-supplementary.pdf]

Supplementary Table S1. Matching table between PHD food groups and KHEI components

Matching level	PHD Food Group	KHEI Component	Notes / Differences
Direct match	Whole grains	Mixed grains intake	- PHD: absolute intake (g/day)<br>- KHEI: proportion of mixed/whole grains ≥0.3 serving/day
	Fruits (all fruits)	Total fruits intake / Fresh fruits intake	- PHD: all fruits<br>- KHEI distinguishes fresh vs. juice
	Vegetables (excluding starchy)<br>: dark green vegetables, red and orange vegetables, other vegetables	Total vegetables intake<br>- Vegetables excluding Kimchi and pickled	- PHD: total vegetables<br>- KHEI separates Kimchi/pickled
	Dairy foods<br>: Whole milk or derivative equivalents	Milk and milk products intake	- Clear correspondence (different units: g/day vs servings/day)
Partial match	Protein sources<br>: Red meat, White meat, Poultry, Fish, Eggs, Legumes, Nuts/seeds	Meat, fish, eggs, and beans intake (combined)	- PHD: separate recommendations;<br>- KHEI: aggregated indicator
	Added sugars	% energy from sweets and beverages	- Not identical<br>- PHD: added sugars (g/day)<br>- KHEI: % energy and includes sweets & beverages
	Added fats<br>: Saturated fats	% energy from saturated fatty acids	- PHD: absolute intake (~6.8g/day)<br>- KHEI: % of energy (<7%)
	Added fats<br>: Unsaturated oils	% energy from fat (total)	- Partial overlap<br>- PHD promotes unsaturated fats<br>- KHEI monitors total fat (15–30% of energy)
No direct equivalent	Tubers or starchy vegetables	-	- Included in PHD (50 g/day) but not separately assessed in KHEI
	Tree nuts, Palm oil, Lard/tallow (restricted)	-	- Present in PHD, absent in KHEI
	-	Sodium intake	- KHEI only (≤2000 mg/day); not included in PHD food groups
	-	Have breakfast / Energy intake (EER balance)	- KHEI only; not part of PHD
